# The use of nerve growth factor in herpetic keratitis: a case report

**DOI:** 10.1186/1752-1947-1-124

**Published:** 2007-10-31

**Authors:** Cellini Mauro, Leonetti Pietro, Campos C Emilio

**Affiliations:** 1 University of Bologna, Department of Surgery and Transplant "A. Valsalva", Ophthalmology Service, Bologna, Italy

## Abstract

**Background:**

We evaluate the role of nerve growth factor (NGF) eye drops to treat a herpetic corneal ulcer resistant to systemic and local acyclovir treatment in an HIV-positive patient.

**Case Presentation:**

A 68 year old HIV-positive male presented with a herpetic corneal ulcer which was treated unsuccessfully with acyclovir. Acyclovir sensitivity of herpes simplex virus was tested with a dye uptake assay and we found that the herpes simplex virus isolated was resistant to acyclovir. We started eye drop therapy with NGF and the corneal herpetic ulcer healed in 23 treatment days.

**Conclusion:**

The case presented here is the first described in the literature in which a herpetic corneal ulcer was successfully treated with NGF. We recommend that trials of NGF therapy in herpetic keratitis should be carried out on a larger number of acyclovir resistant cases.

## Background

Herpes simplex virus (HSV-1) infections of the eye, and in particular keratitis, are quite frequent in people with acquired immunodeficiency syndrome [1] and are generally treated with acyclovir. Recently, there has been a progressive increase in the number of cases resistant to acyclovir [2] due to the development of viral strains resistant to the drug and the appearance of a gene tk mutation [3]. Experimental studies have recently demonstrated that nerve growth factor (NGF) is able to block HSV-1 reactivation [4] and is also able to repair neurotrophic corneal ulcers [5]. On the basis of this, we decided to use NGF eye drops to treat a corneal herpetic ulcer in an HIV-positive patient, where the herpes simplex virus was resistant to systemic and local acyclovir treatment.

## Case presentation

**Figure 1:**
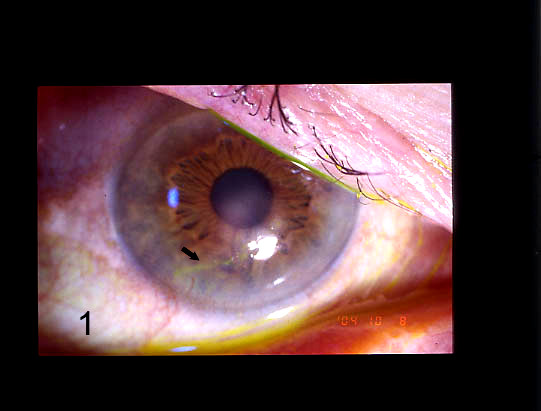
Corneal herpetic ulcer (black arrow) before treatment with nerve growth factor eye drops.

A male patient, aged 68 years, was referred to us in October 2004 with a corneal herpetic ulcer in his right eye. The ulcer had appeared after an influenza vaccination. The anamnesis showed that the patient was HIV-positive without signs of AIDS and with a CD4 count of 496/mm^3^. Furthermore he was not on Highly Active Anti-Retroviral Treatment (HAART). We treated the patient with oral acyclovir, 800 mg five times daily, and topical acyclovir ointment four times daily. After 10 days of treatment no clinical improvement was observed (Fig. 1). We collected a sample of corneal cells that was found to be positive for HSV-1. The sensitivity of the isolated HSV-1 to acyclovir was tested using a dye uptake assay that measured the quantitative cytopathic effect [6] and we found that HSV-1 isolates were resistant to acyclovir.

On the basis of the persistence of the corneal herpetic ulcer we decided to discontinue the topical and systemic acyclovir therapies and to trial topical treatment with NGF eye drops. Before treatment the patient was informed of the procedures and the aim of the therapy and he signed a written consent form. The institutional ethics committee of the S. Orsola-Malpighi Hospital also approved the study.

Treatment consisted of murine NGF purified from sub maxillary glands and produced in the laboratories of the Institute of Neurobiology & Molecular Medicine (CNR-EBR, Roma, Italy).

**Figure 2:**
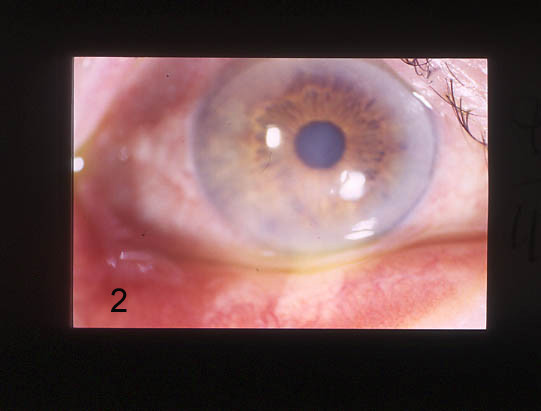
Healing of corneal herpetic ulcer after treatment with nerve growth factor eye drops.

We administered NGF eye drops (10 μg NGF dissolved in 50 μL saline solution and 0.9% sodium chloride) every two hours in the inferior conjunctival fornix until the ulcer was healed, which occurred after 23 days of treatment (Fig. 2). After healing, we continued treatment for another 15 days, reducing the administration of eye drops to just 4 times daily. A year after the treatment with NGF eye drops, the patient was reviewed and there was no sign of recurrent keratitis

## Conclusion

This case illustrates the appearance of HSV-1 viral strains resistant to acyclovir [2]. Acyclovir is a potent and highly selective antiviral nucleoside analog, which remains one of the standard treatments of HSV infections in both immunocompetent and immunocompromised hosts. To become active and inhibit viral DNA synthesis acyclovir must be phosphorylated to acyclovir monophosphate by specific HSV-1 thymidine kinases (tk) and then phosphorylated to acyclovir triphosphate, the active form of acyclovir able to inhibit viral DNA-polymerases, by host cell kinases.

Previous studies indicate that antiviral resistance develops at a stable low rate in pools of HIV. Acyclovir-resistance is based principally on three different mechanisms [7]. Two mechanisms involve a deficiency of viral tk genes, the third shows drug resistance with normal expression of tk. These mutants have either an altered tk enzyme or a mutation in their DNA polymerase gene [7].

NGF is a member of the neurotrophin gene family which also includes neurotrophin 3 (NT-3), neurotrophin 4/5 (NT-4) and brain-derived neurotrophic factor (BDNF). Neurotrophins exert their biological functions by binding to high affinity transmembranous receptors belonging to the trk family of receptor tyrosin kinase including trkA, trkB and trkC and p75 [8].

TrkA is the receptor of NGF and consists of a 140 KD molecule, which modulates not just neuronal survival but also the migration and proliferation of other cell lines [8].

NGF is a polypeptide that is essential for the survival and growth of sympathetic and sensorial system neurons and for CNS neuronal differentiation [8]. The production of NGF and the ubiquitous presence of its specific receptors have also been demonstrated in the ocular surfaces [9]. The mechanism of action of NGF on the ocular surface to heal corneal neurotrophic ulceration is not well defined. It may be that NGF restores a deficit of synthesis or release of endogenous NGF [5]. A direct mechanism may involve the sensory innervation and the proliferation and differentiation of epithelial cells [10]. An indirect mechanism could be related to the increase of those neuropeptides that promote epithelial healing or the release of cytokines.

In vitro and in vivo studies [10] have shown how the depletion of NGF causes a reactivation of HSV-1. This reactivation is the consequence of the loss of balance between the cytokine produced by the corneal epithelial inflammation and the resulting reduction in the NGF-trkA complex signals [4]. Topical administration of NGF has therefore a mainly anti-inflammatory effect blocking the action of the cytokine and restoring the normal transmission of the NGF-trkA complex towards the nerve endings [10].

The case presented here is the first described in the medical literature in which a corneal herpetic ulcer, resistant to antiviral therapy, was treated with NGF. Although the use of NGF for the treatment of this type of disorder is still under debate, we believe that its use in selected cases of herpetic keratitis resistant to acyclovir therapy must be investigated on a larger number of cases to confirm our result.

## Competing interests

The author(s) declare that they have no competing interests.

## Authors' contributions

Mauro Cellini, MD recruited the patient from the Cornea Disease Service of the S. Orsola-Malpighi Hospital; he drafted the manuscript and reviewed the literature. Prof. Emilio Campos examined the patient in the time. Pietro Leonetti reviewed the manuscript.

## Consent

Mauro Cellini, MD (Department of Surgery and Transplant – Ophthalmology Service) examined the patient and received the informed written consent from the patientfor publication of the manuscript and any accompanying images.

## References

[B1] Shuler J Engstrom R Holland G External ocular disease and anterior segment disorders associated with AIDS Int Ophthalmol Clin 1998 29 98 104 10.1097/00004397-198902920-00007 2541098

[B2] Erlich KS Mills J Chatis P Acyclovir-resistant herpes simplex virus infections in patients with the acquired immunodeficiency syndrome N Engl J Med 1989 320 293 296 2536136 10.1056/NEJM198902023200506

[B3] Nugler F Colin JN Aymard M Langlois M Occurrence and characterization of acyclovir-resistant herpes simplex virus isolates: report on a two-year sensitivity screening survey J Med Virol 1992 36 1 12 1315366 10.1002/jmv.1890360102

[B4] Kriesel JD The roles of inflammation, STAT transcription factors, and nerve growth factor in viral reactivation and herpes keratitis DNA and cell biology 2002 21 475 481 10.1089/10445490260099773 12167251

[B5] Bonini S Lambiase A Rama P Caprifoglio G Aloe L Topical treatment with nerve growth factor for neurotrophic keratitis Ophthalmology 2000 107 1347 1351 10.1016/S0161-6420(00)00163-9 10889110

[B6] McLaren C Ellis MN Hunter GA A colorimetric assay for the measurement of the sensitivity of herpes simplex viruses to antiviral agents Antiviral Res 1983 3 223 234 10.1016/0166-3542(83)90001-3 6320716

[B7] Andrei G Snoeck R De Clercq E Esnouf R Fiten P Opdenakker G Resistance of herpes simplex virus type 1 against different phosphonylmethoxyalkyl derivatives of purines and pyrimidines due to specific mutations in the viral DNA polymerase gene J Gen Virol 2000 81 639 648 10675401 10.1099/0022-1317-81-3-639

[B8] Yamamoto M Sobue G Yamamoto K Expression of mRNAs for neurotrophic factors (NGF, BDNF, NT-3, and GDNF) and their receptors (p75NGFR, trkA, trkB, and trkC) in the adult human peripheral nervous system and nonneural tissues Neurochem Res 1996 21 929 938 10.1007/BF02532343 8895847

[B9] Lambiase A Bonini S Micera A Rama P Bovini S Aloe L Expression of nerve growth factor receptors on the ocular surface in healthy subjects and during manifestation of inflammatory diseases Invest Ophthalmol Vis Sci 1998 39 1272 1275 9620090

[B10] Kriesel J Jones B Hwang I Dahms K Spruance S Signal transducers and activators of transcription(STATs) are detectable in mouse trigeminal ganglion neurons J Interferon Cytokine Res 2001 21 445 450 10.1089/107999001750277934 11440643

